# Measuring the impact of an enhanced strategy for daily disinfection in acute-care hospital rooms

**DOI:** 10.1017/ash.2022.142

**Published:** 2022-05-16

**Authors:** Bobby Warren, Aaron Barrett, Amanda Graves, Carly King, Nicholas Turner, Deverick Anderson

## Abstract

**Background:** Enhanced strategies for daily disinfection in acute-care hospital rooms are needed but are poorly understood. **Methods:** We conducted a randomized control trial pilot study in acute-care hospital rooms at Duke University Health System in Durham, North Carolina, comparing the efficacy of a novel EPA-registered quaternary ammonium disinfectant with 24-hour activity, Sani24, to routine daily disinfection. Rooms housing patients on contact precautions were enrolled. In each study room, the bedrails, overbed table, and sink were divided into 2 equal halves, or sides, labeled left and right, with sample areas of 2,000 cm^2^, 1,750 cm^2^, and 400 cm^2^, respectively. Each sample area side was then randomized 1:1 to intervention or control by a coin toss. Sani24 was applied to the surface of each intervention sample side and allowed to air dry. Control sides were left alone. Environmental services (EVS) staff were not involved in the study and were blinded to randomization status. Glogerm dots were applied to all 6 sample-area sides after application of the intervention to measure compliance of daily disinfection by EVS and the removal of the intervention agent. Microbiological samples were taken with sponges premoistened with neutralizing buffer from each sample area side for 6 total samples (3 intervention and 3 control) immediately before and 24 hours following application of the intervention agent. Clinically important pathogens (CIP) were defined as MRSA, VRE, and CRE. The primary outcome was room CFU on study day 1, which was compared using a Wilcoxon rank-sum test. **Results:** In total, 20 patient rooms were enrolled in the study, and 240 samples were obtained from 120 sites (60 intervention and 60 control) from November 2021 to January 2022. Enrolled patients were all on contact isolation and had an active infection; 15 (75%) were bedridden and 8 (40%) were female. On day 0, baseline contamination was similar between study arms: 7,460 (IQR,4,204–16,482) room CFU and 18 samples (30%) harboring CIP in the intervention arm versus 7,273 (IQR, 3,142–21,117) and 15 samples (25%) in the control arm (*P* = .49 and .47, respectively). On day 1, intervention areas had significantly lower CFU at 4,016 (IQR, 2,339–7,358) compared to controls at 6,112 CFU (IQR, 3,484–11,356; *P* = .01). No significant differences were detected between study arms regarding CIP recovery. Glogerm was minimally removed from sample areas (n = 7, 3%), and the result was similar between study arms. **Conclusions:** The use of the quaternary ammonium disinfectant with 24-hour activity on high-touch healthcare surfaces led to reduced contamination over a 24-hour period. Routine daily disinfection compliance by EVS was low since minimal sample areas had Glogerm removed

**Funding:** PDI

**Disclosures:** None

